# The Reliability of Tweets as a Supplementary Method of Seasonal Influenza Surveillance

**DOI:** 10.2196/jmir.3532

**Published:** 2014-11-14

**Authors:** Anoshé A Aslam, Ming-Hsiang Tsou, Brian H Spitzberg, Li An, J Mark Gawron, Dipak K Gupta, K Michael Peddecord, Anna C Nagel, Christopher Allen, Jiue-An Yang, Suzanne Lindsay

**Affiliations:** ^1^Graduate School of Public HealthSan Diego State UniversitySan Diego, CAUnited States; ^2^Department of GeographySan Diego State UniversitySan Diego, CAUnited States; ^3^School of CommunicationSan Diego State UniversitySan Diego, CAUnited States; ^4^Department of LinguisticsSan Diego State UniversitySan Diego, CAUnited States; ^5^Department of Political ScienceSan Diego State UniversitySan Diego, CAUnited States

**Keywords:** Twitter, tweets, infoveillance, infodemiology, syndromic surveillance, influenza, Internet

## Abstract

**Background:**

Existing influenza surveillance in the United States is focused on the collection of data from sentinel physicians and hospitals; however, the compilation and distribution of reports are usually delayed by up to 2 weeks. With the popularity of social media growing, the Internet is a source for syndromic surveillance due to the availability of large amounts of data. In this study, tweets, or posts of 140 characters or less, from the website Twitter were collected and analyzed for their potential as surveillance for seasonal influenza.

**Objective:**

There were three aims: (1) to improve the correlation of tweets to sentinel-provided influenza-like illness (ILI) rates by city through filtering and a machine-learning classifier, (2) to observe correlations of tweets for emergency department ILI rates by city, and (3) to explore correlations for tweets to laboratory-confirmed influenza cases in San Diego.

**Methods:**

Tweets containing the keyword “flu” were collected within a 17-mile radius from 11 US cities selected for population and availability of ILI data. At the end of the collection period, 159,802 tweets were used for correlation analyses with sentinel-provided ILI and emergency department ILI rates as reported by the corresponding city or county health department. Two separate methods were used to observe correlations between tweets and ILI rates: filtering the tweets by type (non-retweets, retweets, tweets with a URL, tweets without a URL), and the use of a machine-learning classifier that determined whether a tweet was “valid”, or from a user who was likely ill with the flu.

**Results:**

Correlations varied by city but general trends were observed. Non-retweets and tweets without a URL had higher and more significant (*P*<.05) correlations than retweets and tweets with a URL. Correlations of tweets to emergency department ILI rates were higher than the correlations observed for sentinel-provided ILI for most of the cities. The machine-learning classifier yielded the highest correlations for many of the cities when using the sentinel-provided or emergency department ILI as well as the number of laboratory-confirmed influenza cases in San Diego. High correlation values (r=.93) with significance at *P*<.001 were observed for laboratory-confirmed influenza cases for most categories and tweets determined to be valid by the classifier.

**Conclusions:**

Compared to tweet analyses in the previous influenza season, this study demonstrated increased accuracy in using Twitter as a supplementary surveillance tool for influenza as better filtering and classification methods yielded higher correlations for the 2013-2014 influenza season than those found for tweets in the previous influenza season, where emergency department ILI rates were better correlated to tweets than sentinel-provided ILI rates. Further investigations in the field would require expansion with regard to the location that the tweets are collected from, as well as the availability of more ILI data.

## Introduction

### Overview

Surveillance systems that are in place by the Centers for Disease Control and Prevention (CDC) and through state surveillance have the potential to reduce morbidity and mortality due to a disease and to improve health; however, their usefulness has not been established [1]. Traditionally, surveillance for disease incidence and prevalence is ongoing and systematic, generally relying on laboratory-confirmed cases, as reported by a clinical physician, laboratory, or emergency department. Between the reporting of cases and compiling of data into surveillance reports, a time delay of 1 to 2 weeks is often present, impacting the response of health departments to a possible outbreak.

The Internet’s potential as a source of public health information has not been overlooked in the past decade. It has been hypothesized that the routine use of informal electronic information, typically user-generated, can reduce the time needed to recognize an outbreak, prevent governments from suppressing outbreak information, and facilitate public health interventions [2]. With the increasing popularity of social media websites and people publicly sharing many aspects of their days, syndromic surveillance systems, which rely on the use of real-time data in order to provide quick analysis and feedback for a potential outbreak, now have a new source of data [3]. Infodemiology, where user-generated data on Internet-based venues has allowed for the possibility to mine, aggregate, and analyze text to inform public health practitioners and public policy, is an emerging field that has applications toward disease outbreak detection [4]. Infoveillance, where information gleaned from infodemiology is used as a method of surveillance [4], can be used to enhance syndromic surveillance and can be applied to influenza activity.

Among vaccine-preventable illnesses, seasonal influenza in adults has the greatest impact in the United States [5]. Although it has similar symptoms to the common cold, infection with the influenza virus can lead to symptoms ranging in severity and can lead to death in susceptible persons [6]. Financially, the annual national economic burden of influenza attributable to adults can amount to US $83.3 billion according to a study from 2007 [7]. While the ever-changing nature of influenza viruses enables new sources of the flu season every year, predictions of the onset of infections and the number of people affected are nearly impossible to make with traditional surveillance methods. Through the implementation of a supplementary surveillance tool focused on real-time trends gathered from social media and the fast release of that data, public health agencies may be better prepared and able to stifle a potentially debilitating outbreak in a community.

### Related Work

More recent disease surveillance websites are Web application hybrids that are capable of mining, categorizing, filtering, and visualizing epidemic information while using geographic information systems (GIS) in real time so that delays are minimized and updates are constant [8-14]. HealthMap, organized by the Children’s Hospital of Boston, has between 1000 and 150,000 users daily and provides real-time updates for public health reports related to all types of outbreaks across the world in many languages [2]. Social media on the Internet has also identified foodborne-illness outbreaks faster than traditional methods as many of those affected opt to not seek medical attention and instead post their symptoms online [15]. Google Flu Trends collects the 50 million most common search queries in Google as they relate to flu symptoms, remedies, and complications and compares them with the CDC’s reported national influenza-like illness (ILI) rates [16]. The benefits of using the Internet are multiple as these Internet tools could aid public health officials to underscore the importance of vaccination and prevention measures, or guide physicians in their medical decision making [2]. However, the lack of specificity of signals, noisy data, false reports, and unusual events like drug recalls or popular cold or flu remedies can overload the tool with irrelevant data that can lead to inaccuracy during analysis [2].

Twitter, a microblogging site where users generate tweets, or texts of 140 characters or less, has already shown its value in forecasting box-office revenues, earthquake reporting, meme tracking, large-scale fire emergencies, downtime on services, live traffic updates, national moods, currency trading [17], and even election results [18]. The real-time updates on Twitter are useful for a variety of fields—whether to increase knowledge, predict consumer trends, or to determine what users are discussing in general. For example, researchers at the University of Michigan were able to use Twitter as a tool to understand the effects of a migraine in real-time by collecting tweets and categorizing them by prevalence, life-style impact, linguistic, and timeline of the self-reported migraine headache, finding that the study avoided memory bias and experimenter-induced error, and highlighted migraine colloquialisms as they related to modern characteristics and descriptions used by migraine sufferers [19].

Multiple studies have been done to find correlations between tweets and ILI data, however the searches tend to be very wide. One study analyzed over 500 million tweets from an 8-month period and found that tracking a small number of flu-related keywords and combinations of keywords allowed forecasting of future rates with a 95% correlation [20]. Signorini et al also found that Twitter can be used descriptively, as a way to ascertain users’ interests and concerns related to influenza, and can capture real-time disease activity [21]. During the influenza A (H1N1) pandemic, hundreds of thousands of tweets were collected in the United Kingdom over a period of 24 weeks to search for symptom-related statements [22]. The method proved to be inexpensive as well as timely by utilizing a stream of data created within only a few hours whereas traditional surveillance would take 1 to 2 weeks to release a report; however, it was determined that it would be necessary to separate media hype and discussion from reporting of actual flu cases if the goal is to use Twitter as a predictive tool for influenza [22]. Chew and Eysenbach reached similar conclusions after performing content analysis from tweets during the 2009 H1N1 outbreak and found that over 90% of their tweets were linked to mainstream and local news websites, but the proportion that were linked to more opinion-based or experience-based sites (blogs, social networks, web pages) also increased over the time of collection [23].

### Objectives

This study builds on previous exploratory research conducted by the Department of Geography at San Diego State University that demonstrated that the content of social media messages and searches was correlated with actual surveillance reports of influenza in the 2012-2013 US influenza season [24]. The objectives of this study were threefold. The first objective was to investigate the ability to improve the correlation of Twitter social media content with traditional sentinel ILI surveillance reports by using a machine-learning classifier and keyword-based search techniques to filter tweets to make them more “valid”. Second, we sought to compare mentions of influenza in social media content to emergency department ILI records, and third, to do a small pilot study comparing tweets related to influenza to laboratory-confirmed influenza cases in San Diego, California. Unlike previous research with Twitter and influenza surveillance, our study is unique in that we compared ILI rates from specific cities to tweets that contained the word “flu” that originated from that respective city, thereby focusing on city-specific correlations. By comparing tweets from a city with the city-specific ILI rates, we are able to view trends in the spread of influenza on a much smaller scale than in previous studies.

## Methods

### Data Collection

Using a geo-targeted social media search tool created by Tsou et al [18], information mining can be conducted in conjunction with the Twitter Search Application Programming Interface (API). With over 200 million active users, Twitter is a large resource of publicly available data in the form of millions of tweets. By first specifying a keyword, the research group’s frameworks in combination with the Twitter API yield a Microsoft Excel spreadsheet of tweets that originate from within a certain geographical location (determined by the user’s global positioning system coordinates, if enabled, or listed hometown) and are associated with the keyword either through the text of the tweet, or the username. The spreadsheet also includes additional data include time of tweet creation, location of origin, who the tweet was directed to if it was part of a conversation, the number of followers, and people following the user who tweeted, as well as the number of total tweets of that user. Of interest to this study were the tweet text and the geographic location of the tweet was posted.

Based on our previous study indicating that non-retweets and tweets without a URL containing the keyword “flu” were much more highly correlated with sentinel influenza surveillance than other words such as “influenza” [24], tweets that contained the keyword “flu” were collected and aggregated once every 7 days starting on August 25, 2013 and ending on March 1, 2014. Tweets were collected from users who resided within a 17-mile radius from the center of 11 different cities (Boston, Chicago, Cleveland, Columbus, Denver, Detroit, Fort Worth, Nashville-Davidson, New York, San Diego, and Seattle). Tweets were collected from a 17-mile radius as it was the minimum distance between two neighboring cities, thus ensuring there were no tweets that could have overlapped in their city of origination. These cities were chosen for their availability of sentinel influenza-like illness (ILI) surveillance data either from the city or county health department. An influenza-like illness is defined as a fever equal to or greater than 100° F and a cough and/or sore throat in the absence of a known cause other than the influenza virus. The ILI is reported as the percent of patients seen for ILI symptoms compared to all patient visits for the week [25]. Because the CDC does not report ILI data below the state level, ILI reports were found on either county or city level health department websites and for San Diego, through a contact at the County of San Diego Health and Human Services Agency. For a subset of five cities (Boston, Chicago, Cleveland, Columbus, and San Diego), both sentinel ILI and emergency department ILI were collected.

At the end of the collection period, 159,802 tweets contained the word “flu” and were used for filtering and analysis. Depending on when ILI data became available by city, the focus was on tweets from Week 40 in 2013 through Week 9 in 2014 (the week starting on September 29, 2013 to the week ending on March 1, 2014), as determined by the CDC’s Morbidity Mortality Weekly Report (MMWR). Correlations and significance of correlation values between the weekly number of yielded tweets and the weekly sentinel ILI or emergency department ILI as reported by the corresponding city or county department were calculated.

### Analysis

Pearson’s correlation coefficients for the association between week-specific tweet volume and influenza-like illness rates were calculated in R (R Foundation for Statistical Computing, Vienna, Austria, version 3.0.0) for each of the cities for tweets containing “flu.” Tweets were also subdivided into non-retweets, retweets, tweets without a URL, and tweets with a URL. These categories were not mutually exclusive, for instance, non-retweets could contain tweets that had a URL or tweets without a URL. This was done to determine whether there were higher correlations based on the type of tweet. Pearson’s correlation coefficients were performed as an easy way to compare groups of tweets with the ILI and in order to readily identify tweets that would be most useful for infoveillance in the future. For each week, the tweeting rate, or the number of tweets per 100,000 individuals, in each city was also calculated. To determine the population of each city that tweets were collected from, census tracts whose centers fell within the 17-mile radius from the city center were identified and their population counts summed. “Flu” tweeting rates were compared weekly for each city and visualized through bar graphs that also displayed the reported ILI rate for each week in each city. The goal of scaling by population was to observe if there would be differing trends in flu activity by city.

Separately, a machine-learning classifier was coded in Python (Python Software Foundation, Delaware, USA, version 2.7.6) and its “scikit-learn” software. We used a support vector machine (SVM) classifier to filter out noise from the data set. To train the classifier, we used 1500 randomly sampled tweets containing the keyword “flu” from the 2012-2013 season as inputs. Each of these 1500 training tweets was manually inspected and tagged as valid or invalid according to the likelihood that they indicated actual cases of influenza. This hand-tagged training set was converted to vector representation using their term-frequency-inverse document frequency (TF-IDF) scores, which is a measure of the statistical significance of each term in a text document. These TF-IDF vectors were then input to the SVM for training. Tweets posted by a user whose username contained the word “flu” were removed because they were collected regardless of tweet content and would introduce noise into the sample size. Tweets that were determined to be representative of a user who was likely ill with the flu were labeled as valid, while other tweets that did not score the minimum were classified as invalid and thereby eliminated before conducting correlation analyses. Examples of the types of tweets the algorithm labeled as valid or invalid are listed in Table 1.

To evaluate the classifier, we manually tagged a test set containing 1000 tweets and ran the classifier to get two performance measures: recall, the portion of tweets that were hand-tagged as valid in the test set that were also correctly classified as valid by the classifier, and precision, the portion of classified “valid” tweets that were also manually tagged as valid. The recall for the classifier was calculated to be 0.9369, and the precision was 0.6859. This means that the classifier was able to correctly identify most manually tagged valid tweets as being valid, but it had difficulty identifying invalid tweets and would mark some as valid.

**Table 1:** Examples of valid and invalid tweets from the machine-learning classifier.

Tweet text	Valid or Invalid
“I hate being sick with the flu”	Valid
“Not a good time to be hit by a flu”	Valid
“Been home sick with the flu the last 2 days”	Valid
“Getting my flu shot”	Invalid
“Now it’s my turn to have the stomach flu. Ugh”	Invalid
“Recipes for Foods That Fight The Flu [URL]	Invalid

## Results

### Sentinel-Provided ILI

Weekly ILI rates as reported by sentinel physicians to city and county health departments were available for Boston, Chicago, Cleveland, Columbus, Denver, Detroit, Fort Worth, Nashville-Davidson, New York, and San Diego. Table 2 shows correlation coefficients between the sentinel-provided ILI for each city and the number of weekly tweets containing the keyword “flu” that originated in each city before filtering and after filtering for each of the categories of tweets.

Correlations for each category of tweets (non-retweets, retweets, tweets without a URL, tweets with a URL) with sentinel ILI by city can be seen in Table 2. Correlations in the table that had a significance of *P*<.05 are denoted with an *e* superscript. Denver, Fort Worth, Nashville-Davidson, and San Diego had significant correlations (*P*<.001) for each category, including all tweets. Cleveland and Detroit both had significant correlations for undivided tweets (Column 1) and all categories with the exception of retweets (Column 3). New York was the only city observed to have a significant correlation with all tweets, and all other categories were shown to have insignificant correlations. With the exception of Boston and Denver, non-retweets (Column 2) had higher correlations than retweets. Tweets without a URL (Column 5) also had a higher correlation than tweets with a URL (Column 6) except for Columbus, Detroit, New York, and San Diego. Column 4 displays the Fisher’s *z* transformation *P* values for the comparison of correlations between non-retweets and retweets, while Column 7 contains the Fisher’s *z* transformation *P* values for the comparison of correlations between tweets without a URL and tweets with a URL. Fisher’s *z* transformations were calculated to demonstrate whether there was a difference between categories of tweets.

Table 3 shows the correlations for all tweets, the number of tweets, *P* values for the correlations, and then the same information for the tweets that were labeled as valid by the Python machine-learning classifier. Using the valid tweets, the correlations were greater and more significant in 5 cities than the correlations for all tweets (Column 1). Column 7 in Table 3 contains Fisher’s *z* transformation *P* values for the comparison of correlations between the undivided tweets and valid tweets. With the exception of Cleveland, the differences between correlations were significant (*P*<.001).

**Figure 1:**
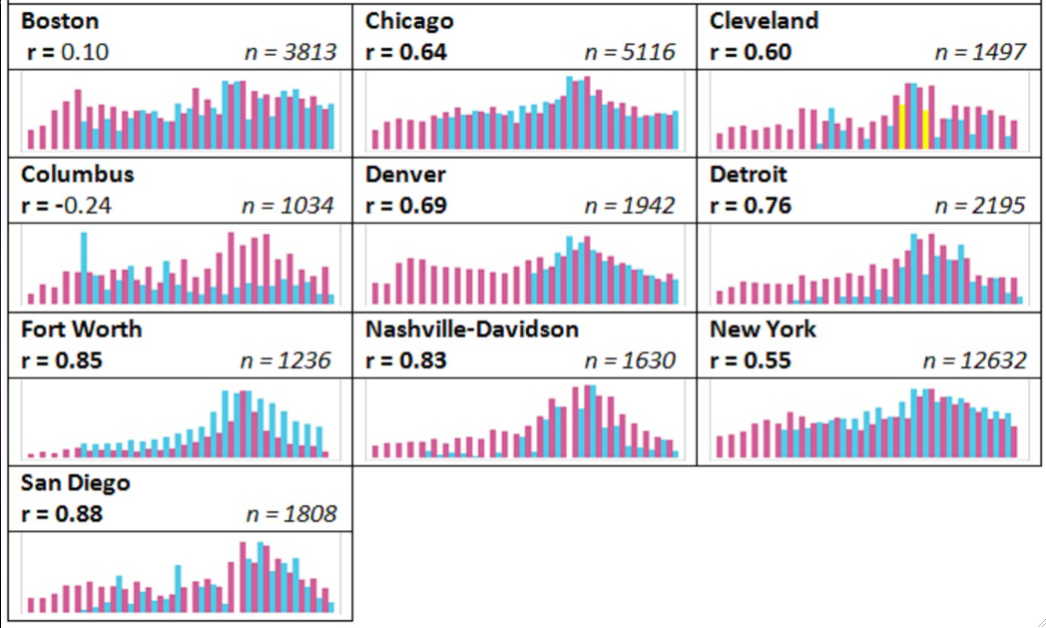
“Valid” Tweet rates per 100,000 versus sentinel-provided influenza-like illness rates by city, 2013-14 influenza season.

Figure 1 shows a visual representation of the weekly tweet rates per 100,000 for the valid tweets in each city alongside the sentinel-provided ILI. The x-axis is the week number, starting at Week 36 and going through Week 9, with two Y-axes: one is tweet rate per 100,000 and the other is the ILI for that week, often reported as a percentage. Correlations from Table 2 are listed and bolded if the significance for the correlation was at *P*<.05, alongside the number of valid tweets for each city. Tweet rates are shown in pink and ILI rates in blue. Yellow bars indicate missing ILI data and were calculated by averaging the ILI rate from the week before and after the week of missing data. To ensure better visualization, maximum ILI rates for each city were rescaled.

Trends in valid tweets containing the word “flu”, or tweets identified to be posted by a user who is likely ill with the flu, and sentinel-provided ILI are displayed in bar charts for each city. Tweeting rates are in pink, ILI rates in blue, and yellow indicates a week during which ILI rates were missing. Both of the tweeting and ILI rates were rescaled for each city in order to show trends on the same scale to account for differences in population. Correlation coefficients between valid tweets and the sentinel-provided ILI rate as well as the total number of valid tweets are listed for each city. Significant correlations (*P*<.05) are bolded.

**Table 2:** Correlations between tweets and sentinel-provided ILI^a^ rates.^b^

	1. All tweets	2. Non-retweets	3. Retweets	4. Fisher’s *z* transformation^c^	5. Tweets without a URL	6. Tweets with a URL	7. Fisher’s *z* transformation^d^	8. Total number of tweets
	*r*	*r*	*r*	*P*	*r*	*r*	*P*	
Boston	−.05	−.19	.08	<.001	.04	−.13	<.001	17,370
Chicago	.33	.50	.04	<.001	.49^e^	.25	<.001	21,655
Cleveland	.63^e^	.74^e^	.42	<.001	.56^e^	.55^e^	.703	6632
Columbus	.01	.05	−.06	.019	−.04	.08	.001	3206
Denver	.76^e^	.64^e^	.74^e^	<.001	.81^e^	.63^e^	<.001	5706
Detroit	.81^e^	.84	.44	<.001	.62^e^	.78^e^	<.001	8417
Fort Worth	.69^e^	.73^e^	.45^e^	<.001	.81^e^	.62^e^	<.001	4755
Nashville-Davidson	.77^e^	.74^e^	.54^e^	<.001	.70^e^	.66^e^	<.001	5805
New York	.44^e^	.42	.39	<.001	.32	.44	<.001	64,340
San Diego	.78^e^	.73^e^	.41^e^	<.001	.69^e^	.73^e^	<.001	8002

^a^ILI: influenza-like illness

^b^Correlation coefficients of all tweets and tweet categories with sentinel-provided ILI rates for each city. Comparisons between tweets and ILI began in Weeks 36-49 (weeks starting September 1, 2013 to starting November 24, 2013) as ILI data became available by city and ended in Week 9 (ending March 1, 2014).

^c^This column displays the *P* values from Fisher’s *z* transformation comparing the correlation coefficients of non-retweets to retweets.

^d^This column displays the *P* values from Fisher’s *z* transformation comparing the correlation coefficients of tweets without a URL to tweets with a URL.

^e^Significant correlation coefficient (*P*<.05).

**Table 3:** Correlations between valid tweets and sentinel-provided ILI^a^ rates.^b^

	1. All tweets, r	2. Number of all tweets	3. *P*-value for all tweets	4. Valid tweets, r	5. Number of valid tweets	6. *P*-value for valid tweets	7. Fisher’s z transformation, *P*
Boston	−.05	17,370	.834	.10	3813	.67	<.001
Chicago	.33	21,655	.139	.64	5116	.002	<.001
Cleveland	.63	7152	.002	.60	1497	.003	.064
Columbus	.01	3288	.978	−.24	1034	.274	<.001
Denver	.76	5706	.003	.69	1942	.009	<.001
Detroit	.81	8417	.001	.76	2195	<.001	<.001
Fort Worth	.69	4755	.001	.85	1236	<.001	<.001
Nashville-Davidson	.77	5805	.001	.83	1630	<.001	<.001
New York	.44	64,340	.047	.55	12632	.01	<.001
San Diego	.78	8002	.001	.88	1808	<.001	<.001

^a^ILI: influenza-like illness

^b^Correlation coefficients between all tweets and valid tweets, as identified by the machine-learning classifier, with sentinel-provided ILI rates for each city. Comparisons between tweets and ILI began in Weeks 36-49 (weeks starting September 1, 2013 to starting November 24, 2013) as ILI data became available by city and ended in Week 9 (ending March 1, 2014).

### Emergency Department ILI Rates

Emergency department ILI rates were available for six cities: Boston, Chicago, Cleveland, Columbus, San Diego, and Seattle. Health departments reported hospital emergency department ILI rates for every city, with the exception of Boston, where data was made available through the Boston Public Health Commission. Table 4 contains the correlations for tweets with the same tweet categories as Table 2: all tweets, non-retweets and retweets, tweets without a URL, and tweets with a URL. In general, non-retweets (Column 2) had higher correlations than retweets (Column 3), and tweets without a URL (Column 5) had higher correlations than tweets with a URL (Column 6) when comparing to the emergency department ILI rates of each city. Fisher’s *z* transformations in Column 4 comparing correlations of non-retweets to retweets and Fisher’s *z* transformations in Column 7 comparing correlations of tweets without a URL to tweets with a URL were significant for all cities for which emergency department ILI rates were available (*P*<.05).

Tweets marked as valid by the classifier were more highly correlated to the emergency department ILI rates than all tweets for all of the cities, as shown in Table 5, with the correlation for tweets and ILI data increasing from .23 (*P*=.41) to .61 (*P*=.02) in Boston alone, and similar increases in correlations observed in the other five cities. The Fisher’s *z* transformation *P* values in Column 7 comparing the correlations of unfiltered tweets and valid tweets to the emergency department ILI rates were all significant (*P*<.001).

**Figure 2:**
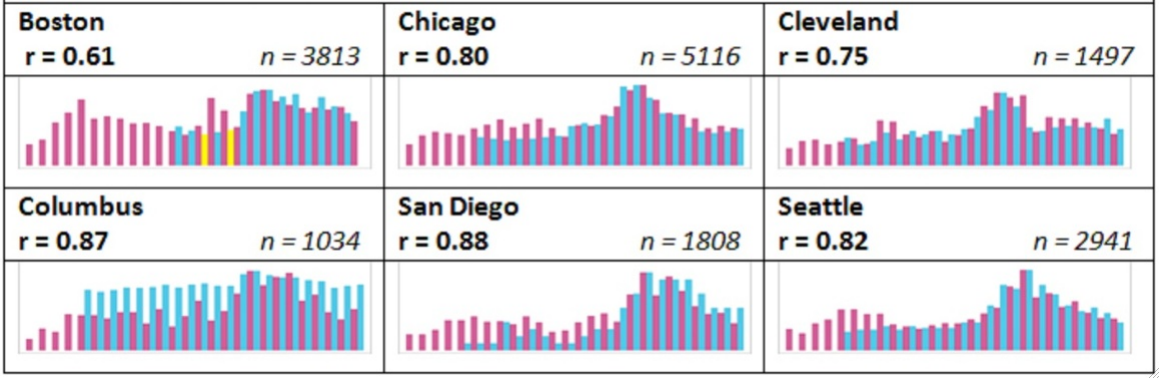
“Valid” Tweet rates per 100,000 versus emergency department influenza-like illness rates by city, 2013-14 influenza season.

Figure 2 shows a visual representation of available emergency department ILI data to rescaled valid tweet rates for each city. The x-axis is the week number, starting at Week 36 and going through Week 9, with two Y-axes: one is tweet rate per 100,000 and the other is the ILI for that week, often reported as a percentage. Pink columns depict tweet rates per 100,000, blue columns show ILI rates, and yellow columns indicate an averaged ILI rate from the week before and the week after a week for which an emergency department ILI rate was not made available.

**Table 4:** Correlations between tweet rates and emergency department ILI^a^ rates by city.^b^

	1. All tweets	2. Non-retweets	3. Retweets	4. Fisher’s z transformation^c^	5. Tweets without a URL	6. Tweets with a URL	7. Fisher’s z transformation^d^	8. Total number of tweets
	*r*	*r*	*r*	*P*	*r*	*r*	*P*	
Boston	.23	.47	−.004	<.001	.03	.41	<.001	17,370
Chicago	.51^e^	.54^e^	.23	<.001	.59^e^	.45^e^	<.001	21,655
Cleveland	.68^e^	.87^e^	.39	<.001	.62^e^	.58^e^	.005	7152
Columbus	.62^e^	.54	.61	.018	.62^e^	.47^e^	<.001	3288
San Diego	.80^e^	.92^e^	.40^e^	<.001	.88^e^	.79^e^	<.001	8002
Seattle	.72^e^	.71^e^	.67^e^	.001	.62^e^	.71^e^	<.001	9735

^a^ILI: influenza-like illness

^b^Correlation coefficients of all tweets and tweet categories with emergency department ILI rates for each city. Comparisons between tweets and ILI began in Weeks 40-41 (weeks starting September 29, 2013 to starting October 6, 2013) as ILI data became available by city and ended in Week 9 (ending March 1, 2014).

^c^This column displays the *P* values from Fisher’s *z* transformation comparing the correlation coefficients of non-retweets to retweets.

^d^This column displays the *P* values from Fisher’s *z* transformation comparing the correlation coefficients of tweets without a URL to tweets with a URL.

^e^Significant correlation coefficient (*P*<.05).

**Table 5:** Correlations between valid tweets and emergency department ILI^a^ rates by city.^b^

	1. All tweets	2. Number of all tweets	3. All tweets	4. Valid tweets	5. Number of valid tweets	6. Valid tweets	7. Fisher’s z transformation
	*r*		*P*	*r*	*r*	*P*	*P*
Boston	.23	17,370	.411	.61	3813	.016	<.001
Chicago	.51	21,655	.017	.80	5116	<.001	<.001
Cleveland	.68	7152	<.001	.75	1497	<.001	<.001
Columbus	.62	3288	.002	.87	1034	<.001	<.001
San Diego	.80	8002	<.001	.88	1808	<.001	<.001
Seattle	.72	9735	<.001	.82	2941	<.001	<.001

^a^ILI: influenza-like illness

^b^Correlation coefficients between all tweets and valid tweets, as identified by the machine-learning classifier, with emergency department ILI rates for each city. Comparisons between tweets and ILI began in Weeks 40-41 (weeks starting September 29, 2013 to starting October 6, 2013) as ILI data became available by city and ended in Week 9 (ending March 1, 2014).

### Laboratory-Confirmed Influenza Cases in San Diego

As a small pilot study, the San Diego Health and Human Services Agency was able to provide the number of laboratory-confirmed influenza cases from Week 40 through Week 9 of the 2013-2014 flu season. Correlations were calculated using the number of weekly tweets in San Diego for all tweets and for each of the tweet categories. Table 6 shows the *r* correlations for all subdivisions of tweets, along with the *P* value for each. All were significant (*P*<.001) with tweets marked as valid by the classifier having the highest correlation value (*r*=.93), followed by non-retweets, tweets without a URL, and all tweets. Retweets had the lowest correlation value at *r*=.40.

**Table 6:** Correlations between tweets and number of laboratory-confirmed influenza cases in San Diego.^a^

All tweets	All tweets	Non-retweet	Non-retweets	Retweets	Retweets	Tweets without a URL	Tweets without a URL	Tweets with a UR	Tweets with a URL	Valid tweets	Valid tweets
*r*	*P*	*r*	*P*	*r*	*P*	*r*	*P*	*r*	*P*	*r*	*P*
.88	<.001	.92	<.001	.40	<.001	.88	<.001	.79	<.001	.93	<.001

^a^Correlation coefficients for all tweets and all categories of tweets, including valid tweets with the number of laboratory-confirmed influenza cases in San Diego starting Week 40 (beginning October 6, 2013) through Week 9 (ending March 1, 2014).

## Discussion

### Principal Findings

This study is a continuation of the exploratory research conducted for the 2012-2013 flu season by researchers at San Diego State University that used Twitter as a possible method for identifying trends in influenza incidence in 11 cities. The specific ILI rates per city are not included in this paper because we wanted to establish the correlations between tweets and ILI rates, regardless of how high or low they were, and not the progression of the spread of influenza in the cities themselves. The 2013-2014 influenza season was less severe than the 2012-2013 influenza season: ILI rates were lower and fewer people were infected with a strain of the influenza virus. Across the 11 cities that tweets were collected from, tweet rates and ILI rates peaked around between Week 50 (ending December 14, 2013) and Week 2 (ending January 11, 2014). Tweets, sentinel, and emergency department ILI rates all followed the same general trend of increasing or decreasing at roughly the same time. Boston was the only city for which the tweet rate peaked before the ILI rate, for both sentinel-provided and emergency department ILI. However, in Boston the correlation between tweet rate and ILI rate was only significant when looking at valid tweets and emergency department ILI (*P*=.02) and other correlations were low and insignificant for that city.

Separate analyses by tweet category suggest methods to improve ILI rate approximation. Non-retweets had higher and more significant correlations than retweets for the majority of the cities, and tweets without a URL also had higher and more significant correlations than tweets with a URL. Non-retweets are completely original tweets and are posted from the user’s location, whereas retweets are the tweets of others re-posted by a user. Even if a tweet is posted from an area outside of collection, retweets can still be acquired because of the location of the user who re-posted it. For this reason, retweets are likely not as reflective of the user’s own health and illness. Tweets with a URL are likely used to share information from a news source or blog and are more probably representative of the user’s opinion or sentiments rather than their actual health condition.

Correlations of tweets with emergency department ILI rates were higher for all of the cities than the correlations of tweets and sentinel-provided ILI rates, with the exception of San Diego. This was observed not only for all tweets, but also for all of the categories of tweets. Emergency department ILI is often reported mandatorily to health departments whereas sentinel-provided ILI is voluntary and based on sentinel physicians within an area. The number of physicians who report weekly can vary widely and lead to inconsistency of rates on a week-by-week basis. Patients who visit a sentinel physician may be more likely to have received an influenza vaccination and so lower ILI rates are reported. Throughout the season, correlations to emergency department ILI activity were very high compared to sentinel-provided ILI activity, though by the end of the study period, the gap in correlations had closed.

Use of the machine-learning classifier yielded the highest correlations for many of the cities when using either sentinel-provided or emergency department ILI activity data, as well as the number of laboratory-confirmed influenza cases in San Diego. We expected to see highest correlations using the valid tweets and the ILI activity, for both sentinel and emergency department ILI rates, because by identifying valid tweets, or ones that were more likely to indicate that the user has an influenza-like illness, much of the noise caused by tweets that are not retweets or do not have a URL can be eliminated. In future infoveillance activities, we recommend the use of non-retweets and tweets without a URL that have been filtered through a machine-learning classifier to improve validity for the highest levels of correlation with sentinel ILI and emergency department ILI findings.

The observation of high correlation values (*r*>.80) and at such high significance (*P*<.001) to laboratory-confirmed influenza cases is another promising aspect of this study. Influenza-like illness rates are based on syndromes and thus provide an idea of the number of illnesses before they can actually be confirmed with laboratory evidence. Nevertheless, the delay that occurs before the reports are released can and does create a large problem for surveillance. Our results show a high correlation between tweets and laboratory-confirmed cases, which may add another source of current information to public health professionals. However, there is growing concern in the field about the effect of large sample sizes on *P* values. It is a possibility that users tweeting about the flu were younger and largely teenagers, who were taken to a physician and tested for the influenza virus because of greater access and their parents taking them, accounting for the especially high *P* value observed in our study between laboratory-confirmed cases and influenza.

An advantage to using social media to survey influenza incidence is that it would quicken response time for public health departments and health care providers. This case study only looked at how well tweets correlated to ILI as reported by emergency departments and sentinel physicians. By observing how both ILI and tweets were increasing together around Weeks 48 through Week 52, a responsive measure to the outbreak could have been instated, whether through notifying neighboring communities of a growing number of flu cases or reminding the population of ways to steer clear of the flu.

### Limitations

The greatest limitation in this study was experienced in the ILI disease reporting surveillance systems. The start dates for the weekly ILI reports varied by city and although some report year-round and others start during MMWR week 40, Denver did not release an ILI rate until MMWR week 49. Reporting by city was also variable with the type of data shared—while some cities had both sentinel-provided and emergency department ILI data, others only had sentinel-provided or, in the case of Seattle, only emergency department ILI data. The optional nature of ILI reporting by sentinel providers meant that cities of similar populations could be gathering data from a differing number of sentinel providers. For instance, Columbus generally had only two or fewer sentinel providers reporting weekly ILI rates and so had unreliable ILI rates for a city of over 800,000 residents. Boston and Chicago also had low correlations between tweets and ILI rates from either source, whether a sentinel provider or emergency department. It is difficult to ascertain why this may be the case because reports from Boston and Chicago did not contain the number of sentinel providers or emergency departments surveyed. As both Boston and Chicago are very large in population size, it may be that there was too much noise in the collected tweets where even tweets that were not posted by news sources were opinion-based, not illness-based, as observed by Chew and Eysenbach [23]. Although correlations improved for both cities when comparing tweets identified as valid to ILI rates, questions remain as to how to utilize Twitter as a tool for Boston and Chicago specifically. One method that would aid our research, as well as surveillance in these cities, would be to perhaps review what qualifies as an ILI and also seek out more sentinel providers willing to report cases, as well as student health centers, as both cities have large student populations who may be accessing their university resources rather than a primary care physician or emergency department. The accuracy of ILI reporting has been brought into question before, but it only increases the need for another method that can be used, such as tweets.

Although information such as username, location, number of followers, number of people being followed, and user profiles can be collected along with the tweet text, demographic information such as age, gender, and race cannot be collected through tweets, making it difficult to determine who is tweeting about the flu and to whom public health efforts should be directed. A total of 31% of Twitter users in 2013 reported their age as between 18-29 years old [26], an age group that can be heavily affected by the flu; although, for many flu strains we are often more concerned about the very young and the elderly. The fact that tweets were highly correlated to ILI surveillance in the 2013-2014 flu season might be due to the fact that a strain of the H1N1 virus was circulating, a strain to which those in 15-24 year age group are considered to be vulnerable. It is hard to know whether or not the correlations would be stronger or weaker if younger and older age groups used Twitter. There was also only one keyword used in this study (“flu”) rather than the large number of keywords used in our first case study. In the previous study, it was found that tweets containing the word “flu” were more highly correlated to ILI rates than tweets that contained the keyword “influenza” or other related terms [24]. However, even with only one keyword, using the machine-learning classifier yielded such high correlations between weekly numbers of tweets and ILI data that it may only be necessary to refine the classifier rather than to include more keywords that would introduce more noise into the data. Other refinements to the classifier would also include fine-tuning the degrees of separation in the tweet. Currently, if a tweet mentions “my sister”, “son”, or “classmate” as having the flu, it is identified as valid. However, if people are tweeting about a celebrity or other popular figure who has the flu, the numbers could be quite skewed. To prevent this issue, more training would be required as a way to modify the algorithm.

### Conclusions

Social media is a growing platform used by millions of people and holds great potential as a resource for public health through infodemiology and infoveillance research. This study demonstrated reproducibility in using Twitter as a supplementary surveillance tool for influenza, as better filtering and classification methods yielded higher correlations than those found for tweets in the previous influenza season. Non-retweets and tweets identified as valid by our machine classifier were both highly correlated to reported ILI rates in many of the cities and specify which tweets should be collected in the future.

Further investigations in this field should include expansion beyond these 11 cities, however more ILI data would need to be available to allow for a possible association to be detected. Our study was restricted as ILI data was available from only 11 cities, but if more cities would publish a weekly ILI rates, either sentinel or emergency room, or both, more refinement could be made in our methods and more knowledge obtained for the reliability of tweets as an indicator of seasonal influenza trends. Existing traditional influenza surveillance efforts have been long-lasting and well-developed, but if correlations to user-generated data on social media continue to increase through improved methods, a real-time estimate of influenza cases would be valuable not only to public health efforts in containing an outbreak and in predicting ILI rates in real time, but also to the general population vulnerable to illness. More credibility should be given to using Twitter as a supplementary large-scale surveillance tool for identifying the spread of local disease in an effort to detect outbreaks earlier and provide more time for the development and implementation of interventions designed to halt the spread of diseases.

## Abbreviations

API: application programming interface
CDC: Centers for Disease Control and Prevention
ILI: influenza-like illness
MMWR: Morbidity Mortality Weekly Report
SVM: support vector machine
TF-IDF: term-frequency-inverse document frequency score
